# Limited heterogeneity in the T-cell receptor V-gene usage in lymphocytes infiltrating human colorectal tumours.

**DOI:** 10.1038/bjc.1994.211

**Published:** 1994-06

**Authors:** B. Ostenstad, M. Sioud, T. Lea, E. Schlichting, M. Harboe

**Affiliations:** Institute of Immunology and Rheumatology, National Hospital, Oslo, Norway.

## Abstract

**Images:**


Br. J. Cancer (1994), 69, 1078-1082 © Macmillan Press Ltd., 1994

### Limited heterogeneity in the T-cell receptor V-gene usage in lymphocytes infiltrating human colorectal tumours

B. Østenstad[1], M. Sioud[1], T. Lea[1], E. Schlichting[2] & M. Harboe[1]

[1]Institute of Immunology and Rheumatology, National Hospital, Oslo, Norway; [2]Department of Surgery, Ullevaal Hospital, Oslo, Norway.

**Summary** The presence of T lymphocytes in solid tumours may reflect an ongoing immune response against the transformed cells. We have used polymerase chain reaction (PCR) technology to investigate the T-cell receptor variable-region gene (V-gene) usage in freshly isolated tumour-infiltrating lymphocytes (TILs) to look for a possible oligoclonality of T cells in the tumour area. We used 19 different Vβ-family-specific primers. Peripheral blood lymphocytes and lamina propria lymphocytes from the same patients were also tested by PCR. Our results demonstrate a limited heterogeneity in the V-gene usage of TILs from seven patients with colorectal cancers, suggesting a local antigen-driven immune response at the tumour site.

The existence of tumour-specific antigens has long been questioned, but recently the first tumour rejection antigen was identified and cloned from a human malignant melanoma (van der Bruggen *et al.*, 1991). Several groups have demonstrated autologous tumour-reactive, MHC-restricted tumour-infiltrating T lymphocytes (TILs) derived from malignant melanomas (Herin *et al.*, 1987; Itoh *et al.*, 1988), and similar results have been obtained with TILs from head and neck cancers (Yasumura *et al.*, 1993) and ovarian carcinomas (Ioannides *et al.*, 1993). *In vivo*, TILs appear to be more effective in adoptive immunotherapy than autologous peripheral blood lymphocytes (PBLs) (Rosenberg *et al.*, 1988), suggestive of a primed population in the tumour area. Few studies are available on TILs from colorectal carcinomas, but a recent report provides evidence for a specific, MHC-restricted immune response to autologous, human colon carcinomas (Hom *et al.*, 1993). Furthermore, the degree of lymphoid infiltration in such tumours is strongly correlated with the prognosis (Svennevig *et al.*, 1984; Jass, 1986), suggesting that patients can raise a significant immune response against these tumours.

Finally, gene transfection studies in murine models have shown that provision of local help in the form of cytokines (Fearon *et al.*, 1990; Golumbek *et al.*, 1992) can induce tumour rejection both locally and distantly, and can even enable the immune system to reject a later challenge with unmodified (parental) tumour cells.

Thus, a specific immune response can be mounted against at least some tumours, although insufficient or abrogated in most cases. Conventional phenotyping of freshly isolated TILs has shown that the infiltrating cells are predominantly T cells, which are activated and express the memory phenotype ($CD45R0^+$) more often than peripheral blood lymphocytes from the same patient (Topalian *et al.*, 1987; Østenstad *et al.*, 1994).

If T cells do recognise and react to tumour-specific antigens, a clonal expansion of the tumour-reactive T cells is expected to be an early event. A clonal expansion of this kind will be characterised by restricted T-cell receptor (TCR) variable region gene (V-gene) usage.

In the majority of T cells the TCR is composed of an α/β-heterodimer, each chain containing a variable and constant region. During T-cell development the genes coding for the variable regions are created by rearrangement of alternative gene segments. The functional β-chain gene, for example, is created by recombination of one of each of at least 106 variable (V), two diversity (D) and 13 joining (J) gene segments (combinatorial diversity) (Mak, 1993). Imprecise junction of the different segments (junctional diversity) and the addition of nucleotides not encoded by either gene segment (N-region diversity) greatly increase the variability of the gene product and together with the α-chain constitute the unique T-cell receptor. The diversity is concentrated in the putative antigen-binding CDR3-equivalent region, corresponding to the D and J regions (and a small part of the 3′-terminal V regions). The V segments are evolutionarily related, and can be grouped together as about 20 gene families.

Preferential expression of certain Vβ-families in the tumour area would be suggestive of a clonal expansion of antigen-specific T cells. Such Vβ restriction has been described in many non-malignant disorders, such as allograft rejections (Ibrahim *et al.*, 1993) and autoimmune diseases (Sioud *et al.*, 1992; Davies *et al.*, 1993). Limited heterogeneity has also been demonstrated in IL-2-expanded TILs (Karpati *et al.*, 1991; Morita *et al.*, 1992). However, the Vβ repertoire is influenced by mitogen stimulation *in vitro* (Wong *et al.*, 1993), and the culture conditions may favour the selective outgrowth of certain subpopulations with minor representation *in vivo*. Thus, a better image of the *in situ* Vβ repertoire can be obtained by testing the freshly isolated lymphocytes. A few groups have demonstrated restricted Vβ-gene usage in freshly isolated TILs from malignant melanomas (Nitta *et al.*, 1991; Ferradini *et al.*, 1993) and hepatocellular carcinomas (Weidmann *et al.*, 1992), while other reports conclude that the immune response to tumour is extremely diverse, with no preferential usage of any gene (Ferradini *et al.*, 1992).

We have investigated the Vβ-gene usage of freshly isolated TILs derived from seven different colorectal tumours using polymerase chain reaction (PCR) and 19 Vβ-family-specific primers, and compared this with T cells from PBLs from the same patients. We have also analysed lymphocytes from the laminal propria (LPLs) in the unaffected resection margin in four of these patients.

### Materials and methods

### Isolation of cells

Peripheral blood and surgical specimens were obtained from seven patients with primary adenocarcinoma of the colon or the rectum.

The lymphocyte preparations were obtained as described previously (Østenstad *et al.*, 1994). Briefly, the tumours were minced and digested enzymatically in complete medium (RPMI-1640 with 25 mM HEPES buffer and 10% fetal calf serum, containing 0.05% Collagenase D, 0.002% DNAse

Correspondence: B. Østenstad, Institute of Immunology and Rheumatology, Fr. Quamsgt. 1, 0172 Oslo, Norway.
Received 13 October 1993; and in revised form 8 February 1994.

(Boehringer Mannheim Biochemica, Germany) and 5 mM calcium chloride. The resultant crude suspension was filtered through a fine nylon mesh (80 μm) to exclude undigested fragments, washed twice in Hanks' balanced salt solution (HBSS), and passed over a discontinous density gradient (Percoll, Pharmacia, Uppsala, Sweden), collecting the lymphocytes from the 60%/30% interface and discarding the debris and dead cells at the top.

One million cells from each compartment were frozen as pellets for later extraction of total RNA.

### *Phenotyping*

Freshly isolated lymphocytes were suspended in cold HBSS containing 0.02% sodium azide in V-bottom plates. Fluorescein-conjugated monoclonal antibodies were added in 1:20 dilutions for staining 30 min on ice. The following antibodies were used: UCHT-1 (anti-CD3), MT310 (anti-CD4), DK25 (anti-CD8), HD37 (anti-CD19), UCHL-1 (anti-CD45R0), ACT-1 (anti-CD25), HLA-DR CR3/43 (reacting with the common MHC class II β-chain) (all from Dako, Glostrup, Denmark), and 2H4 (anti-CD45RA) and NKH-1 (anti-CD56) (from Coulter Immunology, Hialeah, FL, USA).

Analysis was performed using a FACScan flow cytometer (Beckton Dickinson, Mountain View, CA, USA) and LYSYS II software.

### *Preparation of RNA, cDNA synthesis and polymerase chain reaction (PCR) amplification*

Total RNA was prepared using guanidium isothiocyanate–phenol–chloroform extraction (Chomczynski & Sacchi, 1987). cDNA was synthesised from RNA as described previously (Sioud *et al.*, 1991). The same amount of cDNA from each sample was used as template for PCR amplification using a panel of 19 family-specific oligonucleotides and Cβ-primers with sequences as previously described (Sioud *et al.*, 1991).

The scope of this investigation was a non-quantitative search for the relative overrepresentation of certain Vβ-families within each tumour, and each sample therefore served as its own control. However, in order to be sure that all the different reactions were set up with the same accuracy, we also performed co-amplification of the constantly present Cα-segment in a few samples. The sense and antisense Cα-primers used were as described previously (Sioud *et al.*, 1991).

Thirty to forty amplification cycles were performed using a DNA thermal cycler (Perkin Elmer, Cetus). Each cycle consisted of denaturation at 92°C for 1 min, annealing at 56°C for 1½ min and extension at 72°C for 2½ min. The PCR products were separated on a 2% agarose gel, transferred to nylon membrane, hybridised with 5′ $^{32}P$-labelled internal Cβ-primer and 5′ $^{32}P$-labelled antisense Cα-primer and autoradiographed. The autoradiograms were used to identify the Vβ-Cβ bands at expected sizes (approximately 600 base pairs) and the Cα-bands (approximately 140 bp) (Figure 1) PBL and TIL cDNAs from the same patients were always prepared, amplified and run in parallel.

**Figure 1** Vβ-gene products (Vβ-Cβ) obtained by amplification of cDNA from peripheral blood lymphocytes (PBLs), tumour-infiltrating lymphocytes (TILs) and lamina propria lymphocytes (LPL). The numbers on top refer to the Vβ-family-specific primers used. Co-amplification of the constantly present Cα-segment was performed as a control. The arrows indicate signals detectable in the original films.

### Results

### *Phenotypes*

As reported previously (Østenstad *et al.*, 1993), the infiltrating lymphocytes were predominantly T lymphocytes ($CD3^+$), with equivalent numbers of $CD4^+$ and $CD8^+$ subsets (Table I). There was a significantly lower number of NK cells ($CD56^+$) and conversely a higher number of B cells ($CD19^+$) among TILs. They also appeared activated and expressed the memory phenotype ($CD45R0^+$) more often than PBLs from the same patient, suggestive of a primed population in the tumour area.

### *T-cell receptor Vβ-gene usage*

We found that PBLs from each of our patients expressed most of the 19 Vβ-families, consistent with the expected polyclonality in this compartment. In contrast, the tumour-infiltrating lymphocyte (TIL) preparations showed a marked restriction in the V-gene usage (Figures 1 and 2). In TIL 17 only one Vβ-family was detected, as indicated by the arrow. This is strongly suggestive of an overrepresentation of Vβ3 within this sample. Remarkably, this same family is absent from the corresponding PBLs (Table II). In TIL 20, four families are detected, consistent with an overexpression of these families within the sample.

Our method is only semiquantitative, and comparison between two samples is difficult. However, if we look at

**Table I** Phenotypes of freshly isolated lymphocytes

| | *CD3* | *CD4* | *CD8* | *CD56* | *CD19* | *CD45R0/ CD45RA* | *CD25* | *HLA-DR* |
|---|---|---|---|---|---|---|---|---|
| PBLs | 60 | 42 | 25 | 19 | 7 | 0.8 | 10 | 22 |
| TILs | 58 | 30 | 22 | 5** | 19* | 1.5** | 22** | 34* |

The proportions of different antigens are expressed as a percentage of all mononuclear cells. The tumour-infiltrating lymphocytes (TILs) are significantly different from the peripheral blood lymphocytes (PBLs). *$P < 0.05$; **$P < 0.005$.

**Figure 2** Vβ-gene products obtained by amplification of cDNA from peripheral blood lymphocytes (PBLs) and tumour-infiltrating lymphocytes (TILs) from three patients. The numbers on top refer to the Vβ-family-specific primers used. The autoradiograms were overexposed to visualise all the faint amplifications, and the arrows indicate every detectable signal.

**Figure 3** Vβ-gene products obtained by amplification of cDNA from lamina propria lymphocytes (LPLs). The numbers on top refer to the Vβ-family-specific primers used. The autoradiograms were overexposed to visualise all the faint amplifications, and the arrows indicate every detectable signal.

patient 11, Vβ7 but not Vβ8 is detectable in the TIL preparation, while Vβ8 is more strongly expressed in the peripheral blood from the same patient.

Although only three of the patients (8, 17 and 20) had an obvious oligoclonal pattern in their TIL V-gene usage, the last four patients also demonstrated a limited heterogeneity (Table II).

No single gene family was consistently overrepresented in all the tumours.

Because lymphocytes in the gut mucosa are continuously exposed to antigens, we asked ourselves if the limited heterogeneity might be a common feature of lymphocytes residing in this specialised tissue. To address this question, we tested the TCR V-gene usage of lymphocytes from the LPL in the resection margin, away from the tumour, in four of the patients. An intermediate picture was found, with little more than half the families expressed (Figure 1 and 3). While Vβ-families 11 and 15 were absent from all these samples, family 11 was detected in one matched TIL sample (TIL 16), as well as in another unmatched sample (TIL 11), while 15 was expressed in TIL 21. Four of the families present in TIL 16 lacked the corresponding LPL 16.

This overrepresentation of certain Vβ-families in TILs compared with LPLs is also suggestive of a localised clonal expansion in the tumour area.

### Discussion

Much evidence has accumulated during the past few years supporting the hypothesis of a specific immune response against malignant tumours. However, most studies so far have focused on the functional testing of T-cell cytotoxicity (Herin *et al.*, 1987; Itoh *et al.*, 1988; Yasumura *et al.*, 1993) or cytokine secretion (Belldegrun *et al.*, 1989; Hom *et al.*, 1993).

We have used the sensitive PCR technique to investigate the T-cell receptor variable-region gene (Vβ-gene) usage of TILs obtained from seven patients with colorectal tumours together with PBLs from the same patients. We found a limited heterogeneity in all the tumour samples, although to a variable extent. This PCR method is a semiquantitative approach, and care must be taken in comparing different samples, but there is an obvious overrepresentation of a few families within each TIL sample, as indicated in the figures. The results from the PBL samples indicate that the primers function well, and should be able to pick up every family present. Furthermore, a rather high number of amplification cycles were performed.

**Table II** T-cell receptor V-gene usage

| | *Vβ-families* | | | | | | | | | | | | | | | | | | |
|---|---|---|---|---|---|---|---|---|---|---|---|---|---|---|---|---|---|---|---|
| | *1* | *2* | *3* | *4* | *5* | *6* | *7* | *8* | *9* | *10* | *11* | *12* | *14* | *15* | *16* | *17* | *18* | *19* | *20* |
| PBL 8 | + | + | + | + | + | + | + | + | + | + | + | + | + | + | + | + | + | + | + |
| PBL 11 | + | + | + | + | + | + | + | + | + | + | + | + | | | + | + | + | + | + |
| PBL 16 | + | + | + | + | + | + | + | + | + | | + | + | | + | + | + | + | + | + |
| PBL 17 | + | + | | + | + | + | + | + | + | | + | + | | | + | + | + | + | + |
| PBL 20 | + | + | + | + | + | | + | + | + | + | + | + | + | + | + | + | + | + | |
| PBL 21 | + | + | + | + | | + | + | + | + | + | + | + | + | + | + | + | + | + | + |
| TIL 8 | | | | | + | | | | | | | + | | | | | | | |
| TiL 11 | | | | + | + | | + | | | | + | + | + | | | | + | + | + |
| TIL 16 | | | + | + | | + | | + | + | | + | | | | | + | + | + | |
| TIL 17 | | | + | | | | | | | | | | | | | | | | |
| TIL 20 | | + | + | | | | | | | | | + | | | | + | | | |
| TIL 21 | | | | + | + | | | + | | | | + | | + | + | | + | + | |
| TIL 22 | + | + | + | | | + | | + | | | | + | | | | | + | | + |
| LPL 16 | | + | + | | + | | + | + | | | | + | | | + | + | + | + | + |
| LPL 17 | + | + | + | | | + | + | + | + | | | + | + | | | | + | + | + |
| LPL 21 | | + | + | + | | | + | + | | + | | + | + | | + | + | + | + | + |
| LPL 22 | + | + | + | | + | | + | + | | | | + | + | | | + | + | + | |

(+) indicates the families detected in the original films. PBL, peripheral blood lymphocyte; LPL, lamina propria lymphocyte; TIL, tumour-infiltrating lymphocyte.

The fact that different sets of families predominate in each tumour therefore argue against a biased amplification due to variable quality of the primers. The inter-sample variability may actually serve as internal controls.

That no single family is consistently overrepresented in all the tumours may reflect the complexity of the TCR–antigen interaction. T cells (presumably including TILs) recognise antigens in the form of short peptides presented in the context of MHC molecules on the target cell. These peptides may be fragments of mutated gene products in the tumour cell having a unique sequence. The specific interaction between tumour cells and T lymphocytes therefore depends on the presence of immunogenic peptides, suitable antigen-presenting MHC molecules and the specific T-cell receptor (TCR).

The variable pattern of gene usage may be explained by the accumulation of mutations in colorectal tumours (Fearon & Vogelstein, 1992), which may give rise to a number of hypothetical T-cell antigens. Any antigenic peptide may also be more or less well presented in different patients depending on their HLA type and individual expression of MHC molecules. Thus, several antigen–MHC combinations may be present in one tumour, although the host usually responds to only one of several antigens displayed simultaneously on the tumour cell surface (immunodominant epitopes) (Urban *et al*., 1986).

Finally, both tumour-specific effectors and negative regulatory T-cell clones may coexist in the same tumour, as suggested by specific Vβ-depletion studies (Gelber *et al*., 1992). These different subsets may express receptors belonging to different Vβ-families.

With this complexity in mind, we note that in three out of seven patients there was definitely an oligoclonal pattern, with fewer than five families clearly represented. To the best of our knowledge this is demonstrated here in TILs from colorectal cancers for the first time.

A limited heterogeneity was also noted in the rest of the patients, comparing TILs with PBLs. However, the repertoire in these four patients was nearly as heterogenous as in the four control samples from tumour-free LPLs. We had expected a more polyclonal pattern among LPLs. One possible explanation is that this may be caused by a predominant non-specific inflammatory response in the neighbouring area. In TIL 11 and 16 (and to some extent in TIL 21 and 22) such an inflammatory response may obscure a possible specific oligoclonality. It is noteworthy, however, that in one of the paired LPL/TIL samples (16) Vβ4, Vβ6, Vβ9 and Vβ11 were present in TILs, but not in LPLs. In another matched pair (21) Vβ5 and Vβ15 were present in TILs, while absent in LPLs. This is also suggestive of possible tumour-reactive clones.

Taken together, a limited heterogeneity in the Vβ-gene usage was found among TILs, consistent with an expansion of tumour-reactive clones *in situ*. No single predominant Vβ-family was detected in the present tumours, suggesting the involvement of different antigen–MHC combinations. Sequence analysis of the fine specificity in the junctional region may clarify this point.

To better understand the significance of this apparent restriction, it is necessary to combine this approach of TCR phenotyping with functional studies at the clonal level. Monoclonal antibodies against a number of different Vβ-families are now available. In contrast to the PCR approach, such antibodies can be utilised in the detection and sorting of viable cells for functional studies, and possibly also in future for selection of T cells particularly suited for adoptive immunotherapy. Alternatively, putative suppressor clones can be depleted.

In conclusion, this study supports the hypothesis that tumour-specific T lymphocytes reside and expand in the tumour area, although the response may be directed against different antigens.

This work was supported by the Norwegian Cancer Society.

### References

BELLDEGRUN, A., KASID, A., UPPENKAMP, M., TOPALIAN, S.L. & ROSENBERG, S.A. (1989). Human tumor infiltrating lymphocytes. Analysis of lymphockine mRNA expression and relevance to cancer immunotherapy. *J. Immunol*., **142**, 4520–4526.

CHEN, L.P., ASHE, S., BRADY, W.A., HELLSTRØM, I., HELLSTRØM, K.E., LEDBETTER, J.A., McGOWAN, P. & LINSLEY, P.S. (1992). Costimulation of antitumor immunity by the B7 counterreceptor for the lymphocyte-T molecules CD28 and CTLA-4. *Cell*, **71**, 1093–1102.

CHOMCZYNSKI, P. & SACCHI, N. (1987). Single step method of RNA isolation by acid guanidinium thiocyanate–phenol–chloroform extraction. *Anal. Biochem*., **162**, 156–159.

DAVIES, T.E., CONCEPCION, E.S., BENNUN, A., GRAVES, P.N. & TARJAN, G. (1993). T cell receptor V gene use in autoimmune thyroid disease – direct assessment by thyroid aspiration. *J. Clin. Endocrinol. Metab*., **76**, 660–666.

FEARON, E.R. & VOGELSTEIN, B. (1992). A genetic model for colorectal tumorigenesis. *Cell*, **61**, 759–767.

FEARON, E.R., PARDOLL, D.M., ITAYA, T., GOLUMBEK, P., LEVITSKY, H.I., SIMONS, J.W., KARASUYAMA, H., VOGELSTEIN, B. & FROST, P. (1990). Interleukin-2 production by tumor cells bypasses T helper function in the generation of an antitumor response. *Cell*, **60**, 397–403.

FERRADINI, L., ROMAN-ROMAN, S., AZOCAR, J., AVRIL, M.F., VIEL, S., TRIEBEL, F. & HERCEND, T. (1992). Analysis of T cell receptor-alpha/beta variability in lymphocytes infiltrating a melanoma metastasis. *Cancer Res*., **52**, 4649–4654.

FERRADINI, L., MACKENSEN, A., GENEVÉE, C., BOSQ, J., DUVILLARD, P., AVRIL, M .F. & HERCEND, T. (1993). Analysis of T cell receptor variability in tumor-infiltrating lymphocytes from a human regressive melanoma – evidence for *in situ* T cell clonal expansion. *J. Clin. Invest*., **91**, 1183–1190.

GELBER, C., EISENBACH, L., FELDMAN, M. & GOODENOW, R.S. (1992). T cell subset analysis of Lewis lung carcinoma tumor rejection – heterogeneity of effectors and evidence for negative regulatory lymphocytes correlating with metastasis. *Cancer Res*., **52**, 6507–6515.

GOLUMBEK, P., LAZENBY, A.J., LEVITSKY, H.I., JAFFEE, L.M., KARASUYAMA, H., BAKER, M. & PARDOLL, D.M. (1991). Treatment of established renal cancer by tumor cells engineered to secrete interleukin-4. *Science*, **254**, 713–716.

HÉRIN, M., LEMONE, C., WEYNANTS, P., VESSIÉRE, F., VAN PEL, A., KNUTH, A., DEVOS, R. & BOON, T. (1987). Production of stable cytolytic T cell clones directed against autologous human melanoma. *Int. J. Cancer*., **39**, 390–396.

HOM, S.S., ROSENBERG, S.A. & TOPALIAN, S.L. (1993). Specific immune recognition of autologous tumor by lymphocytes infiltrating colon carcinomas – analysis by cytokine secretion. *Cancer Immunol. Immunother*., **36**, 1–8.

IBRAHIM, S., DAWSON, D.V., ELMOHSEN, M.A., ELSAMANNOUDY, F.A. & SANFILIPPO, F. (1993). Differential expression of T cell receptor-V region determinants on infiltrating T cells in rejecting and nonrejecting human kidney, liver, and heart allografts. *Transplant. Proc*., **25**, 80–83.

IOANNIDES, C.G., FISK, B., POLLACK, M.S., FRAZIER, M.L., WHARTON, J.T. & FREEDMAN, R.S. (1993). Cytotoxic T cell clones isolated from ovarian tumour infiltrating lymphocytes recognize common determinants on non-ovarian tumour clones. *Scand. J. Immunol*., **37**, 413–424.

ITOH, K., PLATSOUCAS, C.D. & BALCH, C.M. (1988). Autologous tumor-specific cytotoxic T lymphocytes in the infiltrate of human metastatic melanomas. Activation by interleukin-2 and autologous tumor cells, and involvement of the T cell receptor. *J. Exp. Med*., **168**, 1419–1441.

JASS, J.R. (1986). Lymphocytic infiltration and survival in rectal cancer. *J. Clin. Pathol*., **39**, 585–589.

KARPATI, R.M., BANKS, S.M., MALISSEN, B., ROSENBERG, S.A., SHEARD, M.A., WEBER, J.S. & HODES, R.J. (1991). Phenotypic characterization of murine tumor-infiltrating T lymphocytes. *J. Immunol*., **146**, 2043–2051.

MAK, T.W. (1993). The T cell antigen receptor enters its second decade. *Scand. J. Immunol*., **38**, 209–211.

MORITA, T., SALMERON, M.A., MOSER, R.P., ROSS, M.I. & ITOH, K. (1992). Oligoclonal expansion of V$\beta$8$^+$ cells in interleukin-2 activated T cells residing in subcutaneous metastatic melanomas. *Clin. Exp. Metastasis*, **10**, 69–76.

NITTA, T., BELL, R., OKUMURA, K., SATO, K. & STEINMAN, L. (1991). T cell receptor V-beta gene expression differs in tumor-infiltrating lymphocytes within primary and metastatic melanoma. *Cancer Res.*, **51**, 5565–5569.

ØSTENSTAD, B., LEA, T., SCHLICHTING, E. & HARBOE, M. (1994). Human colorectal tumour-infiltrating lymphocytes express activation markers and the CD45R0 molecule, indicating a primed population of lymphocytes in the tumour area. *Gut*, **35**, 382–387.

ROSENBERG, S.A., PACKARD, B.S., AEBERSOLD, P., SOLOMON, D., TOPALIAN, S.L., TOY, S.T., SIMON, P., LOTZE, M.T., YANG, J.C., SEIPP, C.A., SIMPSON, C., CARTER, C., BOCK, S., SCHWARTZENTRUBER, D.J., WEI, J.P. & WHITE, D.E. (1988). Use of tumour-infiltrating lymphocytes and interleukin-2 in treatment of advanced cancer. *N. Engl. J. Med.*, **319**, 1676–1680.

SIOUD, M., KJELDSEN-KRAGH, J., QUAYLE, A.J., WIKER, H.G., SØRSKAAR, D., NATVIG, J.B. & FØRRE, O. (1991). Immune response to 18.6- and 30-kDa mycobacterial antigens in rheumatoid patients, and V$\beta$ usage by specific synovial T cell lines and fresh T cells. *Scand. J. Immunol.*, **34**, 803–812.

SIOUD, M., KJELDSEN-KRAGH, J., SULEYMAN, S., VINJE, O., NATVIG, J.B. & FØRRE, O. (1992). Limited heterogeneity of T cell receptor variable region gene usage in juvenile rheumatoid arthritis synovial T cells. *Eur. J. Immunol.*, **22**, 2413–2418.

SVENNEVIG, J.L., LUNDE, O.C., HOLTER, J. & BJØRGSVIK, D. (1984). Lymphoid infiltration and prognosis in colorectal carcinoma. *Br. J. Cancer*, **49**, 375–377.

TOPALIAN, S.L., MUUL, L.M., SOLOMON, D. & ROSENBERG, S.A. (1987). Expansion of human tumor infiltrating lymphocytes for use in immunotherapy trials. *J. Immunol. Methods*, **102**, 127–141.

URBAN, J.L., KRIPKE, M.L. & SCHREIBER, H. (1986). Stepwise immunological selection of antigenic variants during tumor growth. *J. Immunol.*, **137**, 3036.

VAN DER BRUGGEN, P., TRAVERSARI, C., CHOMEZ, P., LURQUIN, C., DE PLAEN, E., EYNDE, B. VAN DEN KNUTH, A. & BOON, T. (1991). A gene encoding an antigen recognized by cytolytic T lymphocytes on a human melanoma. *Science*, **254**, 1643–1647.

WEIDMANN, E., WHITESIDE, T.L., GIORDA, R., HERBERMAN, R.B. & TRUCCO, M. (1992). The T cell receptor Vbeta gene usage in tumour-infiltrating lymphocytes and blood of patients with hepatocellular carcinoma. *Cancer Res.*, **52**, 5913–5920.

WONG, F.S., HIBBERD, M.L., WEN, L., MILLWARD, B.A. & DEMAINE, A.G. (1993). The human T cell receptor V-beta repertoire of normal peripheral blood lymphocytes before and after mitogen stimulation. *Clin. Exp. Immunol.*, **92**, 361–366.

YASUMURA, S., HIRABAYASHI, H., SCHWARTZ, D.R., TOSO, J.F., JOHNSON, J.T., HERBERMAN, R.B. & WHITESIDE, T.L. (1993). Human cytotoxic T cell lines with restricted specificity for squamous cell carcinoma of the head and neck. *Cancer Res.*, **53**, 1461–1468.


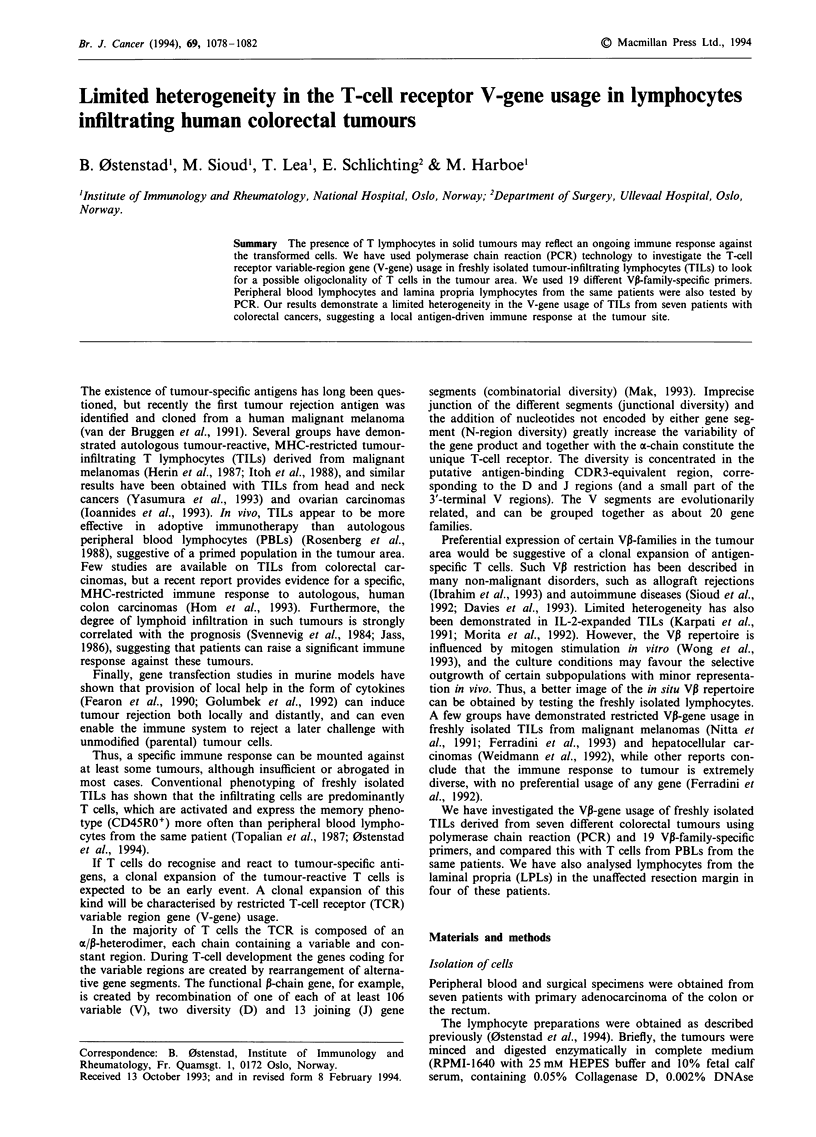

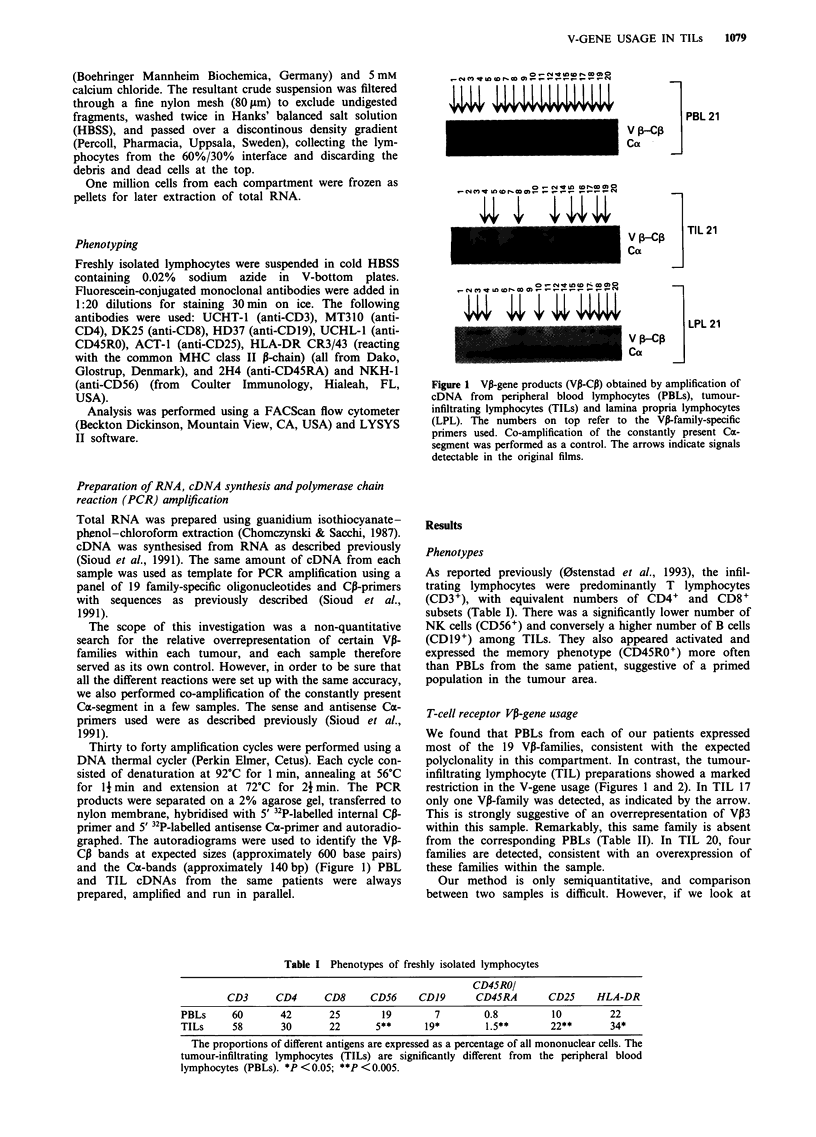

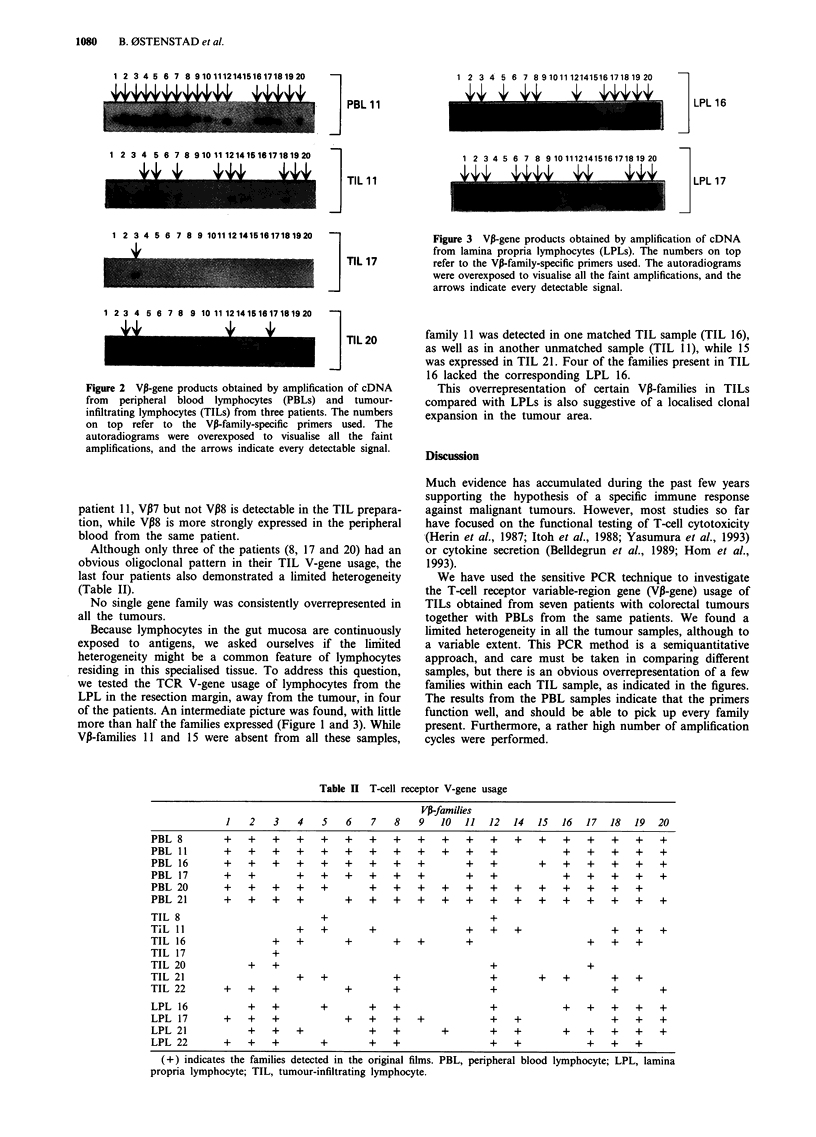

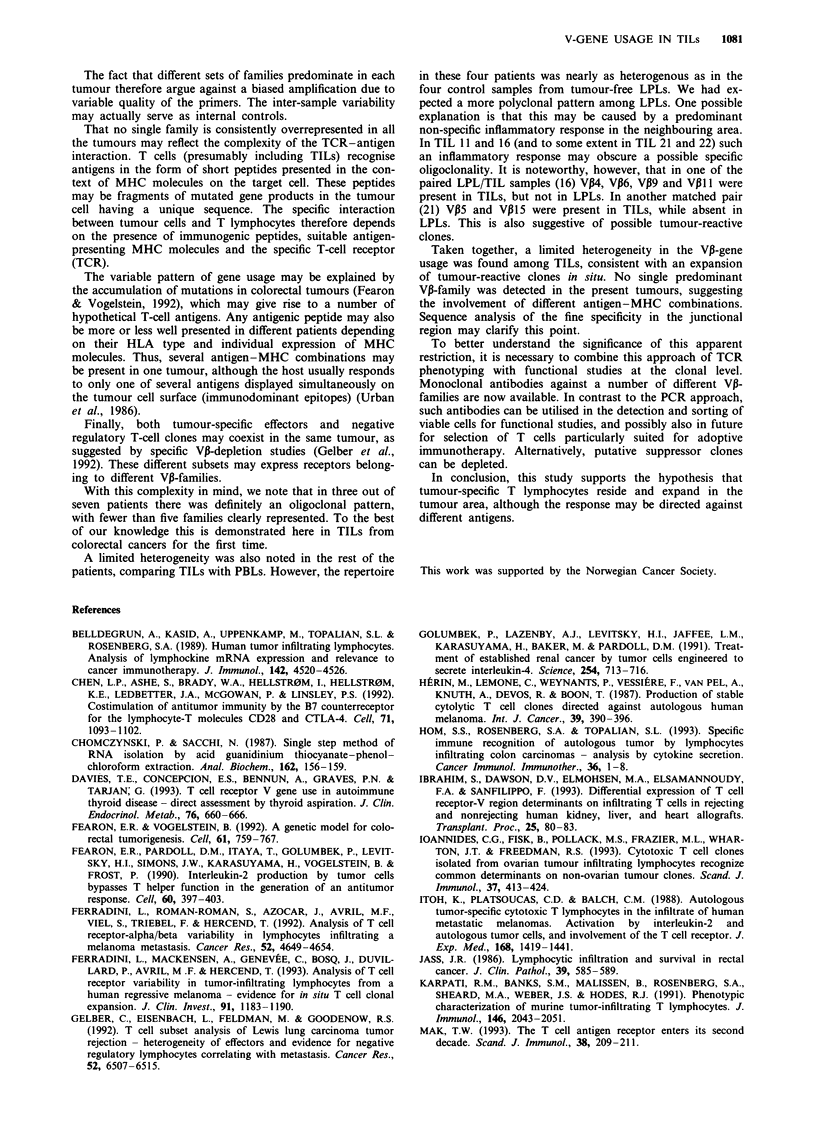

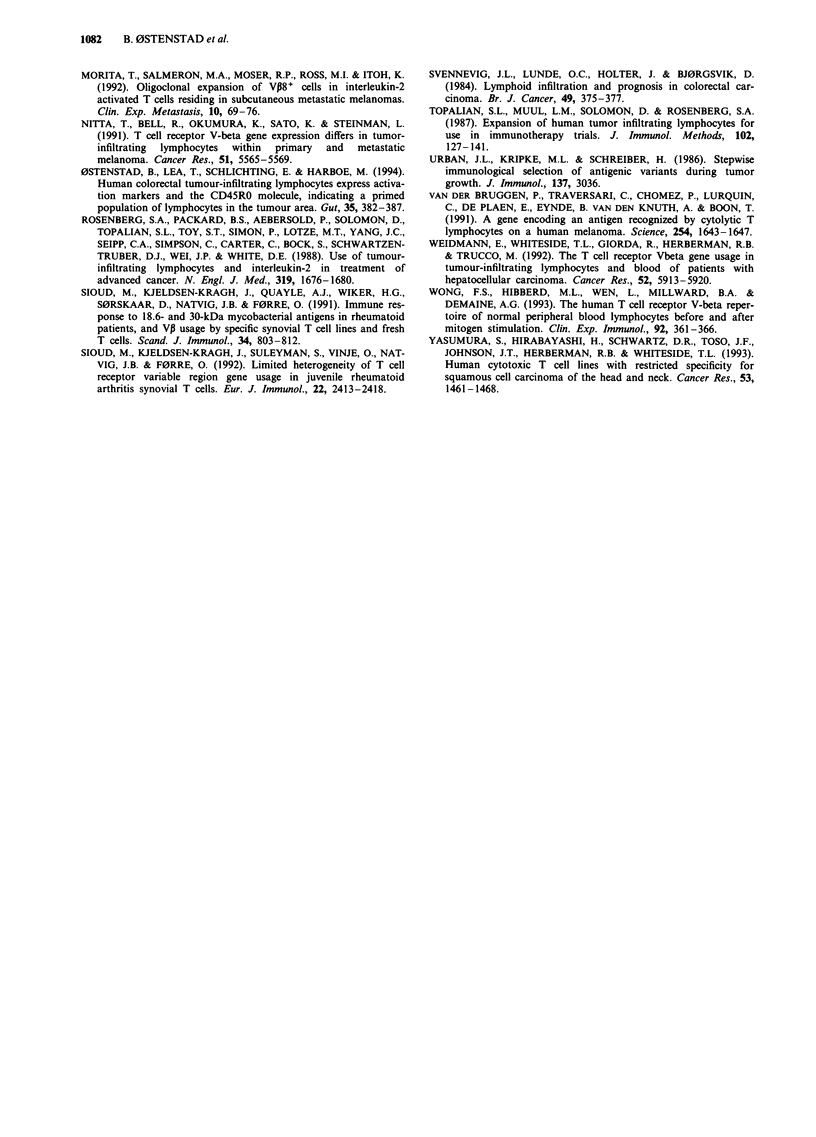


## References

[OCR_00486] Belldegrun A., Kasid A., Uppenkamp M., Topalian S. L., Rosenberg S. A. (1989). Human tumor infiltrating lymphocytes. Analysis of lymphokine mRNA expression and relevance to cancer immunotherapy.. J Immunol.

[OCR_00494] Chen L., Ashe S., Brady W. A., Hellström I., Hellström K. E., Ledbetter J. A., McGowan P., Linsley P. S. (1992). Costimulation of antitumor immunity by the B7 counterreceptor for the T lymphocyte molecules CD28 and CTLA-4.. Cell.

[OCR_00501] Chomczynski P., Sacchi N. (1987). Single-step method of RNA isolation by acid guanidinium thiocyanate-phenol-chloroform extraction.. Anal Biochem.

[OCR_00507] Davies T. F., Concepcion E. S., Ben-Nun A., Graves P. N., Tarjan G. (1993). T-cell receptor V gene use in autoimmune thyroid disease: direct assessment by thyroid aspiration.. J Clin Endocrinol Metab.

[OCR_00518] Fearon E. R., Pardoll D. M., Itaya T., Golumbek P., Levitsky H. I., Simons J. W., Karasuyama H., Vogelstein B., Frost P. (1990). Interleukin-2 production by tumor cells bypasses T helper function in the generation of an antitumor response.. Cell.

[OCR_00512] Fearon E. R., Vogelstein B. (1990). A genetic model for colorectal tumorigenesis.. Cell.

[OCR_00531] Ferradini L., Mackensen A., Genevée C., Bosq J., Duvillard P., Avril M. F., Hercend T. (1993). Analysis of T cell receptor variability in tumor-infiltrating lymphocytes from a human regressive melanoma. Evidence for in situ T cell clonal expansion.. J Clin Invest.

[OCR_00523] Ferradini L., Roman-Roman S., Azocar J., Avril M. F., Viel S., Triebel F., Hercend T. (1992). Analysis of T-cell receptor alpha/beta variability in lymphocytes infiltrating a melanoma metastasis.. Cancer Res.

[OCR_00536] Gelber C., Eisenbach L., Feldman M., Goodenow R. S. (1992). T-cell subset analysis of Lewis lung carcinoma tumor rejection: heterogeneity of effectors and evidence for negative regulatory lymphocytes correlating with metastasis.. Cancer Res.

[OCR_00543] Golumbek P. T., Lazenby A. J., Levitsky H. I., Jaffee L. M., Karasuyama H., Baker M., Pardoll D. M. (1991). Treatment of established renal cancer by tumor cells engineered to secrete interleukin-4.. Science.

[OCR_00555] Hom S. S., Rosenberg S. A., Topalian S. L. (1993). Specific immune recognition of autologous tumor by lymphocytes infiltrating colon carcinomas: analysis by cytokine secretion.. Cancer Immunol Immunother.

[OCR_00549] Hérin M., Lemoine C., Weynants P., Vessière F., Van Pel A., Knuth A., Devos R., Boon T. (1987). Production of stable cytolytic T-cell clones directed against autologous human melanoma.. Int J Cancer.

[OCR_00561] Ibrahim S., Dawson D. V., Elmohsen M. A., Elsamannoudy F. A., Sanfilippo F. (1993). Differential expression of T-cell receptor-V region determinants on infiltrating T cells in rejecting and nonrejecting human kidney, liver, and heart allografts.. Transplant Proc.

[OCR_00570] Ioannides C. G., Fisk B., Pollack M. S., Frazier M. L., Taylor Wharton J., Freedman R. S. (1993). Cytotoxic T-cell clones isolated from ovarian tumour infiltrating lymphocytes recognize common determinants on non-ovarian tumour clones.. Scand J Immunol.

[OCR_00575] Itoh K., Platsoucas C. D., Balch C. M. (1988). Autologous tumor-specific cytotoxic T lymphocytes in the infiltrate of human metastatic melanomas. Activation by interleukin 2 and autologous tumor cells, and involvement of the T cell receptor.. J Exp Med.

[OCR_00582] Jass J. R. (1986). Lymphocytic infiltration and survival in rectal cancer.. J Clin Pathol.

[OCR_00586] Karpati R. M., Banks S. M., Malissen B., Rosenberg S. A., Sheard M. A., Weber J. S., Hodes R. J. (1991). Phenotypic characterization of murine tumor-infiltrating T lymphocytes.. J Immunol.

[OCR_00592] Mak T. W. (1993). The T-cell antigen receptor enters its second decade.. Scand J Immunol.

[OCR_00598] Morita T., Salmeron M. A., Moser R. P., Ross M. I., Itoh K. (1992). Oligoclonal expansion of V beta 8+ cells in interleukin-2-activated T cells residing in subcutaneous metastatic melanoma.. Clin Exp Metastasis.

[OCR_00604] Nitta T., Bell R., Okumura K., Sato K., Steinman L. (1991). T-cell receptor V beta gene expression differs in tumor-infiltrating lymphocytes within primary and metastatic melanoma.. Cancer Res.

[OCR_00610] Ostenstad B., Lea T., Schlichting E., Harboe M. (1994). Human colorectal tumour infiltrating lymphocytes express activation markers and the CD45RO molecule, showing a primed population of lymphocytes in the tumour area.. Gut.

[OCR_00620] Rosenberg S. A., Packard B. S., Aebersold P. M., Solomon D., Topalian S. L., Toy S. T., Simon P., Lotze M. T., Yang J. C., Seipp C. A. (1988). Use of tumor-infiltrating lymphocytes and interleukin-2 in the immunotherapy of patients with metastatic melanoma. A preliminary report.. N Engl J Med.

[OCR_00623] Sioud M., Kjeldsen-Kragh J., Quayle A. J., Wiker H. G., Sørskaar D., Natvig J. B., Førre O. (1991). Immune responses to 18.6 and 30-kDa mycobacterial antigens in rheumatoid patients, and V beta usage by specific synovial T-cell lines and fresh T cells.. Scand J Immunol.

[OCR_00632] Sioud M., Kjeldsen-Kragh J., Suleyman S., Vinje O., Natvig J. B., Førre O. (1992). Limited heterogeneity of T cell receptor variable region gene usage in juvenile rheumatoid arthritis synovial T cells.. Eur J Immunol.

[OCR_00636] Svennevig J. L., Lunde O. C., Holter J., Bjørgsvik D. (1984). Lymphoid infiltration and prognosis in colorectal carcinoma.. Br J Cancer.

[OCR_00641] Topalian S. L., Muul L. M., Solomon D., Rosenberg S. A. (1987). Expansion of human tumor infiltrating lymphocytes for use in immunotherapy trials.. J Immunol Methods.

[OCR_00647] Urban J. L., Kripke M. L., Schreiber H. (1986). Stepwise immunologic selection of antigenic variants during tumor growth.. J Immunol.

[OCR_00658] Weidmann E., Whiteside T. L., Giorda R., Herberman R. B., Trucco M. (1992). The T-cell receptor V beta gene usage in tumor-infiltrating lymphocytes and blood of patients with hepatocellular carcinoma.. Cancer Res.

[OCR_00663] Wong F. S., Hibberd M. L., Wen L., Millward B. A., Demaine A. G. (1993). The human T cell receptor V beta repertoire of normal peripheral blood lymphocytes before and after mitogen stimulation.. Clin Exp Immunol.

[OCR_00669] Yasumura S., Hirabayashi H., Schwartz D. R., Toso J. F., Johnson J. T., Herberman R. B., Whiteside T. L. (1993). Human cytotoxic T-cell lines with restricted specificity for squamous cell carcinoma of the head and neck.. Cancer Res.

[OCR_00653] van der Bruggen P., Traversari C., Chomez P., Lurquin C., De Plaen E., Van den Eynde B., Knuth A., Boon T. (1991). A gene encoding an antigen recognized by cytolytic T lymphocytes on a human melanoma.. Science.

